# Extent of Beta Cell Destruction Is Important but Insufficient to Predict the Onset of Type 1 Diabetes Mellitus

**DOI:** 10.1371/journal.pone.0001374

**Published:** 2008-01-02

**Authors:** David J. Klinke

**Affiliations:** 1 Department of Chemical Engineering, West Virginia University, Morgantown, West Virginia, United States of America; 2 Department of Microbiology, Immunology and Cell Biology, West Virginia University, Morgantown, West Virginia, United States of America; Mayo Clinic College of Medicine, United States of America

## Abstract

**Background:**

Type 1 diabetes mellitus is characterized by an inability to produce insulin endogenously. Based on a series of histopathology studies of patients with recent onset of the disease, it is commonly stated that the onset of clinical symptoms corresponds to an 80-95% reduction in beta cell mass. Motivated by the clinical importance of the degree of beta cell destruction at onset, a meta-analysis was used to determine the validity of this common wisdom.

**Methods and Findings:**

The histopathology results identifying insulin containing islets in patients younger than 20 years of age were extracted from three different studies. The results for 105 patients were stratified by duration of diabetic symptoms and age at onset. Linear regression and a non-parametric bootstrap approach were used to determine the dependence of residual beta cell mass to age at onset. The percentage reduction in beta cell mass was highly correlated (p<0.001) with the age of onset with the greatest reduction in beta cell mass in the youngest patients. As this trend had not been previously observed, an alternative physiology-based model is proposed that captures this age-dependence.

**Conclusions:**

The severity in beta cell reduction at onset decreased with age where, on average, a 40% reduction in beta cell mass was sufficient to precipitate clinical symptoms at 20 years of age. The observed trend was consistent with a physiology-based model where the threshold for onset is based upon a dynamic balance between insulin-production capacity, which is proportional to beta cell mass, and insulin demand, which is proportional to body weight.

## Introduction

Type 1 diabetes mellitus is characterized by an impaired ability to produce insulin due to the progressive and selective destruction of beta cells in the pancreatic islets of Langerhans [1]. The destruction of beta cells is attributed to a variety of environmental and genetic risk factors [2] and results in the inability to maintain glucose homeostasis. While there are promising therapeutics in the drug pipeline (e.g. anti-CD3 [3]), there are currently no treatments available to stop the progressive destruction of insulin-producing beta cells. The standard of care for many patients with Type 1 diabetes mellitus is currently exogenous insulin or insulin analog therapy [4]. The requirement for exogenous insulin, associated with reduced control of glucose homeostasis, results in an increased incidence of significant long-term side effects [5]. Any effort to prolong endogenous insulin production may have a significant impact on the long-term quality of life for patients with Type 1 diabetes mellitus. Clinical management of this disease faces two significant challenges: the increase in incidence of Type 1 diabetes mellitus across the globe [6], [7] and the lack of a cure. Improved understanding of the natural history of this disease underpins possible solutions to these challenges.

One of the persistent challenges with understanding the natural history of Type 1 diabetes mellitus is the inability to observe directly the events in the human pancreas that lead to the onset of hyperglycemia. It is clear that a reduction in endogenous insulin production precipitates the onset of hyperglycemia. Knowledge detailing the pathogenesis in humans is limited to a small number of biopsy studies from individuals with recent disease onset and to data from individuals who died soon after diabetes onset [8]–[11]. Based upon these landmark studies [12], it is a widely held belief that the onset of hyperglycemia occurs when 80–95% of an individual's beta cells are destroyed [13], [14]. The level of destruction may not be so severe as a series of recent studies suggest that impairment in beta cell function may also contribute to the emergence of hyperglycemia [15]–[17].

Quantifying the relative reduction in beta cell mass has important implications for the design of clinical trials to prevent or to improve management of the disease [18]. Given that common wisdom suggests that the reduction in beta cell mass is so severe at onset, one might conclude that the ability to enhance beta cell function or preserve the remaining beta cells would have limited therapeutic potential. In addition, the extent of reduction at onset can influence the debate [19] over the risk versus benefit analysis for novel drugs that prolong endogenous insulin production. Given the clinical importance of this question, the objective of this study was to re-examine the histopathological evidence that has long provided the basis for the conceptual models for the pathophysiology of Type 1 diabetes mellitus. A meta-analysis was used to extract and assess the significance of embedded trends within these landmark studies. It was found that the trends observed within these landmark studies do not fully support the common wisdom that onset of clinical symptoms of Type 1 diabetes occurs when 80–95% of an individual's beta cells are destroyed. An alternative model is proposed where the level of destruction varies with age and depends on the insulin-producing capacity, which is proportional to beta cell mass, and insulin demand, which is proportional to body weight.

## Results

The reduction in beta cell mass for recent onset patients with Type 1 diabetes were extracted from three landmark studies identified in the literature. For the two studies by Foulis and coworkers, the percentage reduction in beta cell mass was based on the ratio of the number of insulin deficient islets to the total number of islets observed following immunohistochemical staining of serial sections of the pancreata from Type 1 diabetic patients [8], [9]. Concurrent observation of insulin-deficient and insulin-containing islets provides an internal control to correct for age-dependent and experimental variability. This calculation assumed that the density of islets in pancreata from Type 1 diabetic patients is representative of age-matched normal controls. To justify this assumption, it is noted that the number of islets and the density of somatostatin-secreting delta cells, glucagon-secreting alpha cells, and other cells of the endocrine pancreas are similar between Type 1 diabetic patients and normal controls [9].

**Figure 1:**
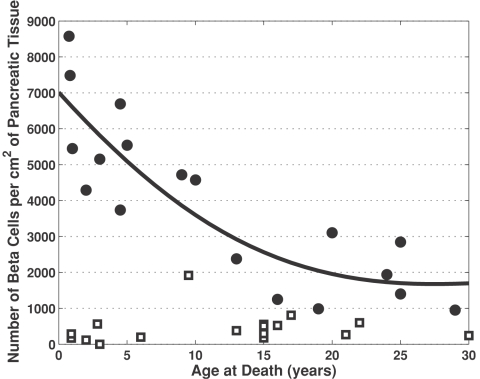
The density of beta cells within a cross-section of a pancreas as a function of age. The density of beta cells were reported for non-diabetic controls (filled circles) and Type 1 diabetics (open squares) reported by Gepts [10]. A non-linear regression was used to estimate the expected density of beta cells in non-diabetic controls as a function of age (solid line). In diabetics, the percentage reduction in beta cell mass is estimated by the ratio of the observed beta cell density in diabetics to the expected density provided by the non-linear regression.

In the study by Gepts [10], the total pancreatic volume and number of beta cells were reported per area of pancreatic tissue for both non-diabetic controls and Type 1 diabetic patients. The calculated reduction in beta cell mass was based on the changes in density of beta cells within pancreata from diabetic patients, as the total pancreatic volume for recent onset patients was reported to be similar to non-diabetic controls. The percentage reduction in beta cell mass was determined based on the ratio of observed density of beta cell to the expected beta cell density based on a patient's age. As shown in Figure 1, non-linear regression (solid line) was used to estimate the density of beta cells in non-diabetic controls (filled circles) as a function of age. In non-diabetic controls, the density of beta cells in the pancreas declines following birth and reaches a plateau at 25 years of age. The density of beta cells for patients diagnosed with Type 1 diabetes mellitus prior to their death (squares) are also shown in Figure 1.

**Figure 2:**
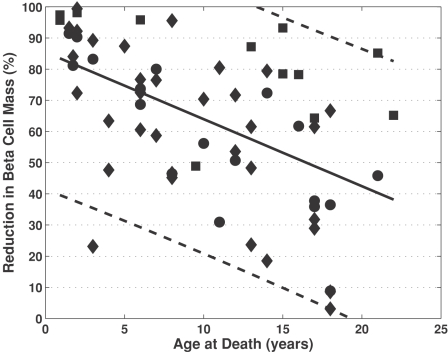
The reduction in beta cell mass is highly variable and depends on age of diagnosis. The data points, shown as black symbols, summarizes the percentage reduction in beta cell mass in 63 subjects that died within three weeks of diagnosis of type 1 diabetes mellitus from three different studies (squares [10], diamonds [9], and circles [8]). The solid line represents the linear regression to the patients with recent onset type 1 diabetes mellitus. The 95% confidence intervals that enclose the population of recent onset type 1 diabetics patients are shown by the dashed lines.

The reduction in beta cell mass is highly variable among a subset of 63 patients identified to have died within three weeks of diagnosis for Type 1 diabetes mellitus (recent onset patients) and shows dependence on age of diagnosis (see Figure 2). The linear regression results suggest that a greater reduction in beta cell mass is required for the onset of hyperglycemia in younger patients compared to older patients. Quantitatively, linear regression of the recent onset patients resulted in an intercept and slope of 85.5 (95% C.I. = 75.3 to 95.2) percent and −2.15 (95% C.I. = −3.02 to −1.30, *p*<0.001) percent year^−1^, respectively. The subset of recent onset patients exhibited considerable inter-individual heterogeneity whereby 95% of the population falls within ±45% of the average reduction in beta cell mass for a given age (dashed lines).

**Figure 3:**
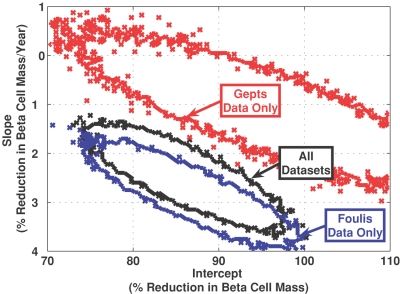
A computational Bayesian estimate of the two-dimensional 95% confidence regions for the linear regression parameters. The confidence regions for the slope and intercept were estimated from the Gepts data (red) [10], Foulis et al. data (blue) [8], [9], and combined data (black). The curves indicate the parameter values that are enclosed within 95% of the posterior probability density function. The confidence regions associated with the combined data is a non-linear combination of the confidence regions associated with the Gepts data only and the Foulis et al. data only.

Individually, the sets of data analyzed in this study exhibit known heterogeneity due to different measurement techniques and potential patient selection bias. Thus, it is important to assess how combining these individual sets of data influences the linear regression results. A computational Bayesian approach, based upon a Markov chain Monte Carlo (MCMC) algorithm, was used to estimate how the uncertainty in the linear regression parameters changed upon combining sets of data [20]. A 2-dimensional (2-D) marginal posterior density (2-D MPD) was used to estimate the confidence regions of the linear regression parameters. In Figure 3, a single contour line indicates the 2-D MPD region within the parameter space that contains 95% of the total marginal posterior density (i.e., the 95% confidence region). The MCMC algorithm was repeatedly applied to the Gepts data only (red curve), the Foulis data only (blue curve), and the combined data (black curve). This repetitive approach was used to assess how the estimates of the parameter values change when considering a composite data set derived from individual sets of data.

To quantify the effect of the inclusion criteria for the analysis, a series of linear regressions were applied to a subset of patients who died prior to the cutoff. The cutoff was varied between one week and 312 weeks after diagnosis. These linear regression results for various cutoff times are shown in Table 1. The linear regression of the entire population of 105 patients also exhibits the same general trends, where the extent of reduction in beta cell mass at onset decreases with age. The slope and intercept for the linear regression relationship of the entire population were determined to be −1.10 (95% C.I. = −1.86 to −0.35, *p = *0.001) percent year^−1^ and 79.8 (95% C.I. = 70.8 to 89.4) percent, respectively.

**Table 1:** The linear regression parameters and corresponding 95% confidence intervals reported as a function of change in the cutoff date.

Cutoff Weeks	N_data_	Slope	Bootstrap 95% C.I.	Intercept	Bootstrap 95% C.I.
1	30	−2.58	(−1.36 to −3.54)	90.09	(74.84 to 100.73)
2	47	−2.59	(−1.66 to −3.27)	91.91	(79.64 to 99.99)
3	63	−2.15	(−1.28 to −2.88)	85.46	(73.58 to 94.25)
6	72	−1.92	(−1.06 to −2.65)	84.00	(72.15 to 93.14)
10	78	−2.05	(−1.21 to −2.74)	84.03	(72.51 to 92.55)
15	87	−1.45	(−0.84 to −1.98)	79.11	(67.82 to 88.25)
26	95	−1.46	(−0.87 to −1.97)	80.34	(69.78 to 89.14)
52	97	−1.42	(−0.84 to −1.94)	80.63	(69.97 to 89.32)
104	103	−1.16	(−0.58 to −1.66)	79.88	(69.28 to 88.22)
312	105	−1.10	(−0.52 to −1.60)	79.81	(69.20 to 88.31)

C.I. = confidence interval

N_data_ = number of patients included in subset

## Discussion

The results from this meta-analysis suggest that clinical presentation of Type 1 diabetes mellitus is correlated with a greater percentage loss in beta cell mass in infants compared to adults. An 85% reduction in beta cell mass in infants can lead to hyperglycemia. In contrast, as little as a 40% reduction by 20 years of age is sufficient to induce hyperglycemia. This meta-analysis also demonstrates the significant heterogeneity in pathophysiology within a population of Type 1 diabetic patients. In older patients, a lower reduction in beta cell mass gives rise to the same clinical phenotype. This trend was also observed qualitatively by Foulis et al. [9]. Given this inconsistency with existing models for the natural history of the disease [2], an alternative theory may be proposed to explain this behavior.

### A Physiology-based Model for Residual Beta Cell Mass at Onset

**Figure 4:**
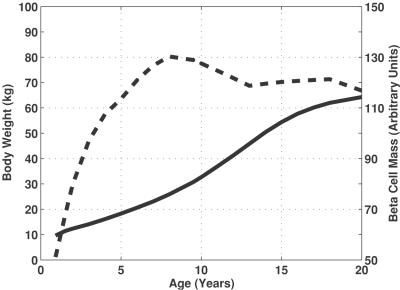
The growth rates for beta cell mass and total body weight exhibit different kinetics. The changes in beta cell mass (dotted line) and body weight in kg (solid line) are shown a function of age. The beta cell mass is the product of beta cell density [10] and volume of the pancreas [10]. The change in body weight as a function of age is an average value from male and female growth charts [25].

Growth of the human body is a dynamic non-linear process where different parts of the body grow at different rates. Of particular relevance to Type 1 diabetes mellitus, body weight changes at a different rate than beta cell mass. One possible explanation for this observed trend in residual beta cell mass at onset could be attributed to the dynamic imbalance between the number of beta cells and the insulin requirements for a growing body. Individually, beta cell density and total volume of the pancreas both vary as a function of age. The age-related growth in total beta cell mass, as shown in Figure 4 (dotted line), can be estimated by the product of the beta cell density and the total volume of the pancreas [10].

In addition, changes in body weight can influence how insulin regulates glucose homeostasis. Cellular response to insulin is proportional to the concentration of insulin. Concentration is a derived intrinsic variable and defined as the number of proteins per unit of volume. As the body grows, the volume in which proteins are distributed increases. Therefore, the number of insulin molecules must also increase to maintain a constant concentration (i.e., elicit the same biological effect). Insulin synthesis and release by beta cells exhibits a non-linear dependence on glucose concentration [21], [22]. If we assume that the cellular capacity to synthesize insulin does not vary with age, each beta cell is capable of contributing a fixed number of insulin proteins per unit of time. Under normal conditions, beta cells may synthesize only a fraction of their maximum capability. As the number of beta cells decreases, the remaining beta cells may increase their individual contributions to maintain a normal concentration of insulin in the blood. As the beta cell mass progressively declines, individual contributions rise until a maximum level of protein synthesis is reached in the remaining beta cells. A subsequent decline in beta cell mass results in an inability of the body to maintain an appropriate concentration of insulin in the plasma.

**Figure 5:**
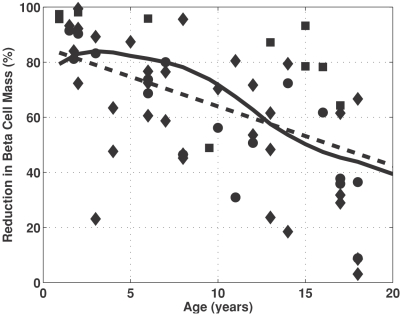
Comparison of the observed reduction in beta cell mass versus the LR and PB models. Comparison of the estimate of excess beta cell mass (solid curve) compared against the trend obtained by linear regression (dotted line) for the measured reduction in beta cell mass in 63 patients that died within three weeks of diagnosis of type 1 diabetes mellitus from three different studies (squares [10], diamonds [9], and circles [8]).

Clinically, metabolic parameters, such as fasting glucose, are observed to increase dramatically at the onset of hyperglycemia [23], [24]. Admittedly, these studies were performed in high risk populations and may not generalizable to the entire Type 1 diabetes patient population. By focusing on patients who died within three weeks of onset, we hypothesize that the onset of hyperglycemia is a titration of the beta cell mass required to maintain glucose homeostasis for a given body weight. A quantitative relationship can be created to predict the “excess” beta cell mass (EBCM(t)) as a function of age. The “excess” beta cell mass corresponds to the reduction in beta cell mass that is required before hyperglycemia occurs. This relationship is derived from a dynamic mole balance between the source of insulin, which is proportional to beta cell mass (BCM_Total_(t)), and sinks for insulin, which are proportional to body weight (BWt(t)) [25] (see Appendix S1). Quantitatively, this relationship can be expressed as:
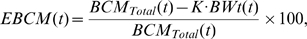
(1)where *K* is a proportionality constant that provides an estimate of the required beta cell mass per kilogram of body weight. Using the patient results for the recent onset group, the value for the parameter *K* was estimated to be 499 units of BCM kg^−1^ (95% C.I. = 458 to 605). The resulting relationship for EBCM(t) as a function of age is shown in Figure 5 (solid line). The trend obtained by linear regression (dotted line) and the observed reduction in beta cell mass in pancreata obtained from the subset of recent onset patients are also shown for comparison.

Qualitatively, the EBCM relationship exhibits a similar dependence with age, as the youngest patients exhibited the greatest potential reduction in beta cell mass. The trend also declines with increasing age, resulting in only a 40% excess in beta cell mass by the age of 20. In other words, the beta cell mass initially grows at a faster rate relative to the whole body. The overall beta cell mass plateaus while the overall body weight steadily increases through 20 years of age. The net result of the different growth dynamics is that the “excess” beta cell mass declines with age. Qualitatively, the observed reduction in beta cell mass in recent onsets diabetics appears to be normally distributed around the predicted “excess” beta cell mass. In the youngest patients (age <three years), the predicted “excess” beta cell mass appears to be lower that the values observed experimentally. In these younger patients, bias may exist due to aggressive autoimmune attack on the beta cells coupled with an inability to identify diabetic symptoms in patients at this young age.

**Figure 6:**
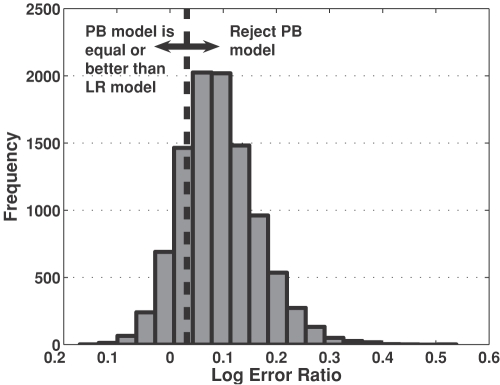
Frequency of values for LER determined via bootstrap resampling with replacement of the residuals. The dotted line corresponds to the AIC threshold for rejecting the PB model. Rejection of the PB model is not statistically significant as the associated p-value equals 0.19.

Quantitatively, objective criteria have been established for selecting among competing models that describe the same phenomenon. In this study, the two competing models are non-nested and include the model based upon the linear regression results (LR model) and physiology-based model (PB model). Bootstrap resampling with replacement (N = 10,000) was used to estimate the confidence level for model discrimination based on the Akaike Information Criterion (AIC). At each bootstrapped sample, the best fit values for the model parameters were used to calculate the LER for the LR and PB models. A histogram showing the distribution of bootstrapped LER values is shown in Figure 6. The AIC for the LR and PB models is calculated to be 0.0317 (dotted line). Based on the experimental data, the LER is estimated to be 0.1245 and suggests that the PB model should be rejected. As shown in Figure 6, the confidence level (p-value) for rejecting the PB model is 0.19 and does not reach the level of statistical significance. Therefore, the PB model is indistinguishable from the LR model in describing the reduction in beta cell mass as a function of age. Moreover, the PB model provides a mechanistic explanation for the observed trends.

**Figure 7:**
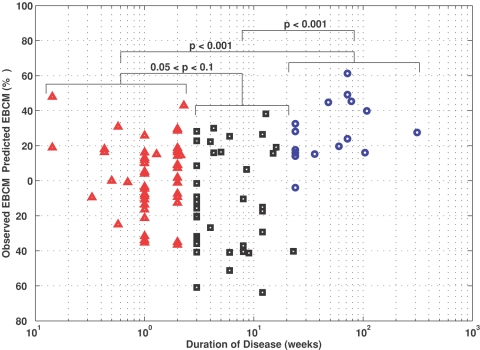
The variance in PB model residuals depends on duration of diabetic symptoms. The observed reduction in beta cell mass for all patients minus the predicted excess beta cell mass matched for age plotted as a function of duration of diabetic symptoms. Patients are grouped into three subsets. The first subset is patients who died within three weeks of diagnosis (red triangles). The second subset is patients who died between three and 23 weeks following diagnosis (black squares). The third subset is patients who died greater than 23 weeks following diagnosis (blue circles). Patients who died greater than 23 weeks following diagnosis exhibited a greater reduction in beta cell mass compared to other groups. The p-values indicate the level of confidence associated with the hypothesis that the mean residual values are different between the two groups.

The duration of diabetic symptoms is also thought to influence the residual beta cell mass. Using the predicted “excess” beta cell mass, each of the 105 patients can be compared against the remaining beta cell mass in an average recent onset patient, matched for age. In Figure 7, the difference between the observed reduction in beta cell mass and predicted “excess” beta cell mass for each of the 105 patients are plotted against the duration of disease. In this figure, the patient population is subdivided into three groups based upon the duration of disease: duration <3 weeks (red triangles), 3<duration<23 weeks (black squares), and duration >23 weeks (blue circles). In the recent onset group, the residual values appear to distribute normally around the predicted values. In addition, patients with duration greater than 23 weeks exhibited, on average, a greater loss in beta cell mass than predicted by the “excess” beta cell mass relationship (p<0.001). While not statistically significant, patients with an intermediate duration (3<duration<23 weeks) exhibited, on average, a lower loss in beta cell mass than predicted by the “excess” beta cell mass relationship (0.1<p<0.05). This observation may be due to patient selection bias. Alternatively, this behavior could be attributed to a regrowth in beta cell mass following initiation of treatment. However, a regrowth of beta cell mass in humans has never been observed following onset of clinical symptoms.

### Potential Limitations and Sources of Bias

A common concern with meta-analyses is the selective bias that exists in the published literature towards data that authors, reviewers, and editors find sufficiently novel to publish [26]. Three additional studies were identified in the literature [11], [27], [28] but were excluded from the analysis due to their small sample sizes (N = 1). In the histopathology studies used, inadvertent bias could have been introduced due to patient selection. The patients selected for these studies had died primarily of diabetic ketoacidosis. The reported pancreata may come from more severe cases and may be an overestimate of the extent of beta cell reduction, as most newly diagnosed patients with Type 1 diabetes mellitus are successfully managed clinically [29].

This analysis suggests that the duration of diabetic symptoms was insufficiently controlled in previous analyses. Qualitatively, the studies included in this meta-analysis focused on recent onset patients. Unfortunately, there is not a consistent definition of recent onset. By applying a linear regression to all of the reported histopathology results, the residuals were found to be non-normally distributed and heteroscedastic (i.e., the variance in residuals depends on the independent variable, age), suggestive of bias in patient selection. This could have been a contributing factor to the high value reported by Gepts for the observed reduction in beta cell mass in all patients diagnosed with Type 1 diabetes mellitus. This was one of the motivations to restrict the analysis to patients that died within three weeks of diagnosis. The residuals for this subset of patients were normally distributed and homoscedastic (i.e., the variance is independent of the independent variable) (data not shown). A second motivation for restricting the analysis was that the clinical practice for metabolic control may have been different at the time of data collection than the current standard of clinical care. By limiting the patient selection to three weeks following onset, the sample size was still sufficiently large and the contribution of differences in clinical practice could be minimized.

**Figure 8:**
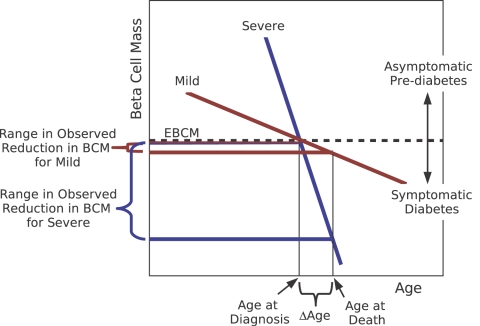
The influence of disease severity on the range of observed reduction in beta cell mass. The diagnosis for Type 1 diabetes occurs once the beta cell mass declines to a point where the excess beta cell mass (EBCM) is depleted (dotted line). The range of observed reduction in beta cell mass is greater in patients with severe autoimmunity to beta cells compared to patients with mild autoimmunity when Δage is greater than zero. As Δage is decreased, the difference in observed reduction in beta bell mass is also decreased.

One may argue that younger patients exhibit an aggressive phenotype compared to older patients and that the linear regression results merely reflect these differences in severity. Prior to onset, patients who will develop Type 1 diabetes remain largely asymptomatic with regard to fasting plasma glucose [23], [24], [30], [31]. Post-onset, the presence of C-peptide, produced in equimolar quantities as insulin, is used as a marker of endogenous insulin production [32], [33]. Following a brief increase post-onset, C-peptide continues to decline in the years following onset. In Figure 8, a diagram is used to illustrate the influence of severity on the potential range of observed reduction in beta cell mass at the time of death. As the difference between the age at diagnosis and the cutoff for age at death is large, the observable range in the remaining beta cell mass gives a lower mean value for an aggressive phenotype than for a mild phenotype. However as the difference between age at diagnosis and cutoff for age at death (Δage) goes to zero, there is no difference in the ranges in beta cell mass for aggressive versus mild phenotypes. This is another motivation for restricting the analysis to patients that died within three weeks of diagnosis. Moreover, if differences in severity exhibited age bias (i.e., younger are severe and older are mild), one would expect that as Δage is reduced the measured slope should increase (i.e., becomes less negative). This trend is not observed in Table 1. Alternatively, a fast rate of decline in C-peptide in younger patients compared to older patients could be attributed to volume of distribution effects as younger patients grow at a faster rate than older patients.

In the years since the publication of these landmark studies, a number of criticisms have been raised regarding the methods used in these studies to accurately assess beta cell mass. In the Gepts study [10], a series of non-specific methods were used to identify beta cells and islets within cross-sections of pancreata, as this study was performed prior to the advent of histological techniques for detecting insulin. In comparison to modern immunohistological techniques, it is unknown how sensitive these non-specific methods are in detecting difference in changes in beta cell mass. The 2-D MPD was used to assess this potential source of bias.

As shown in Figure 3, there is greater uncertainty in the selection of the slope and intercept parameters in the Gepts study, due in part to the smaller sample size (N = 12). This is reflected in the greater area enclosed by the 95% confidence region of the 2-D MPD shown in Figure 3 (red line). Conversely, the 95% confidence region is smaller for the data derived from the Foulis studies (blue line) as a larger number of observations were reported (N = 51). In general, the slope and intercept were higher using only the Gepts data compared to the Foulis data only. Interestingly, the 95% confidence 2-D MPD region obtained from the combined data is not a weighted linear combination of the individual analysis, as the intercept for the combined data set shifts to lower value relative to either the Gepts or the Foulis confidence regions. This effect may be due to a non-normal distribution in sampling (i.e., bias) inherent in the small size of the Gepts study. In the analysis of the combined data, the Bayesian approach provided similar estimates of the confidence intervals as the Bootstrap approach. As shown in Figure 3, one-dimensional projections of the 2-D 95% confidence regions obtained by a Bayesian analysis were similar to the confidence bounds obtained by the non-parametric Bootstrap approach, shown in Table 1. In both cases, the 95% confidence regions associated with the slope parameter are entirely below -1 and provide support for the conclusion that the reduction in beta cell mass at the onset of clinical symptoms of Type 1 diabetes mellitus is dependent on the age of onset.

In studies reported by Foulis et al. [8], [9], islets were classified as either insulin-positive or insulin-negative. This binary classification approach may provide an overestimate of the remaining beta cell mass, as insulin-positive islets may exhibit a reduced complement of beta cells compared to age-matched controls. Yet as demonstrated by several authors [11], [28], the pattern of beta cell destruction within the pancreas is markedly heterogeneous, whereby pancreatic islets with no beta cell destruction can be located nearby fully destroyed islets devoid of beta cells. This binary classification scheme only overestimates the remaining beta cell mass in islets with an intermediate complement of beta cells. A more quantitative assessment of the beta cell mass would need to account for both changes in beta cell numbers within insulin-positive islets and changes in the distribution of insulin content within the population of islets. In summary, there are potential sources of bias in the original data that may influence the conclusions derived from the data analysis. Limited by these data, corrective action, such as restricting the analysis to data obtained prior to three weeks post onset, was taken to minimize the contribution of sampling bias.

### Conclusions

In summary, the fundamental characteristic of Type 1 diabetes mellitus is the loss of endogenous insulin production. A series of landmark studies attributed this loss of endogenous insulin production to the progressive and selective destruction of beta cells in the pancreatic islets of Langerhans. In contrast to other interpretations of these data, this study explicitly accounted for the duration of diabetic symptoms in determining the extent of reduction in beta cell mass at disease onset. By re-examining the data obtained from the pancreata of patients who died soon after diagnosis, the extent of beta cell death was found to vary with age, whereby a 40% reduction at 20 years of age is sufficient to precipitate clinical symptoms of Type 1 diabetes mellitus. Thus, the statement that an 80–95% reduction in beta cell mass is necessary for the onset of hyperglycemia appeared to be an overestimate for most patients. As an alternative to existing models for the natural history of the disease, the observed age dependence was explained using a model based upon a dynamic mole balance on insulin. This dynamic mole balance included a source for insulin, which is proportional to the beta cell mass, and sinks for insulin, which are proportional to body weight, and captured the observed age-dependence. These results emphasize the opportunity to preserve endogenous insulin production, especially in older patients, if aggressive action is taken once patients present with Type 1 diabetes mellitus. Moreover, these results suggest a more holistic model where autoimmune-mediated destruction of pancreatic beta cells is important but insufficient to reconstruct the natural history of the disease.

## Methods

### Study Selection

The research approach was to apply a meta-analysis to histopathology studies which reported insulin positive cells in pancreatic biopsies from patients exhibiting symptoms of Type 1 diabetes mellitus. Three studies were identified where histopathologies of the endocrine pancreas were quantitatively reported for a group of young patients. All of the patients were under the age of 25 and had died from primarily diabetic ketoacidosis [8]–[10]. In total, 105 patients were included from these three studies.

### Calculation of Beta Cell Mass

Histologically, the total beta cell volume can be estimated by transecting the pancreas and quantifying the area of the transected surface that contains insulin positive cells (i.e., viable beta cells) using immunohistochemical techniques. The total volume (i.e., mass) of the beta cells (Vol_BCM_) is related to the ratio of insulin-positive area (A_Ins+_) to total surface area of the transected pancreas (A_Total_), and the total volume of the pancreas (Vol_Pancreas_) according to the following relationship [34]:
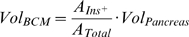
(2)


In a summary of post-mortem results for recent onset patients with Type 1 diabetes mellitus, Gepts [10] and Doniach et al. [35] reported that pancreatic weight did not differ markedly from the expected weight, matched for age. Thus, changes in the total beta cell volume for recent onset patients are assumed to be reflected in changes in insulin-positive area averaged across cross-sectional slices of the pancreas.

### Statistical Analysis of Reduction in Beta Cell Mass

The histopathology results were stratified into different subsets based upon the duration of diabetic symptoms. Patients who had exhibited clinical symptoms of diabetes mellitus for less than three weeks were grouped into a recent onset subset (N_data_ = 63). A linear regression was applied to the recent onset subset to quantify the dependence between percentage reduction in beta cell mass and age at death. Using the linear regression results, a residual value was calculated for each patient to quantify the deviation from the average behavior. A non-parametric bootstrap approach was applied to the population of residual values (N_data_ = 63 or 105) to estimate confidence intervals and significance of the linear regression parameters [36]. A bootstrap sample size (N_boot_) of 10,000 was used to estimate the parameter confidence intervals. A non-parametric bootstrap approach is advantageous as it allows for the adjustment of confidence intervals when the distribution in the dependent variables is not known *a priori*. When the dependent variables are known to exhibit a Gaussian distribution, the bootstrap approach provides a good estimate of the confidence intervals compared against classical statistical techniques, given a sufficiently large bootstrap sample (i.e., N_boot_≥1000) and data sample (i.e., N_data_≥30) [37]. The reported significance corresponds to the probability (*p*) that this relationship does not depend on age. A *p* value of less than 0.05 was considered statistically significant. Repeated analyses were performed by increasing the cutoff date until the entire patient population (N_data_ = 105) was included in the analysis. Tests of significance between the measured reduction in beta cell mass and predicted “excess” beta cell mass was performed using the Behrens-Welch test statistic evaluated against a *t* distribution with the degrees of freedom calculated using the Satterthwaite's approximation [38].

### Statistical Analysis of Data Synthesis

A computational Bayesian approach was used to estimate how the uncertainty in the linear regression parameters changed upon combining sets of data [20]. The uncertainty in the linear regression parameters, as predictors of a given set of data, was represented by a 2-dimensional marginal posterior probability density of the likely parameter values. The 2-D marginal posterior probability densities were calculated using kernel marginalization of the expectation values of the parameters. A 2-dimensional grid of 100×100 kernels (i.e., histogram bins) were created within a range of potential parameter values. A large number of Metropolis-Hasting samples (N_sample_ = 210,000) within a Markov chain Monte Carlo (MCMC) algorithm were used to estimate the expectation values. Lower sampling frequencies also exhibited similar results (data not shown). Prior to testing Monte Carlo samples of parameter space, 5,000 equilibration steps were used to establish the Markov chain. Confidence regions within the parameter space were identified that enclose 95% of the marginal posterior probability densities.

### Model Discrimination

Objective criteria have been established for discriminating between competing models by quantifying the trade-off between model parsimony and model fitness in predicting the dependent variable [39], [40]. The log error ratio (LER) is used to quantify the relative fitness of the competing models in representing the dependent variables.
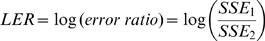
(3)where SSE_i_ is the residual summed squared error for model *i*. Positive values for LER favor the selection of model 2, while negative values favor the selection of model 1. Values for LER around zero suggest that the models are indistinguishable. The Akaike Information Criterion (AIC) has been established for discriminating between competing models [39], [40]. This criterion was established to quantify the trade-off between the relative fitness of competing models (i.e., LER), the number of adjustable parameters (*M*) in a model, and the number of observations of the dependent variable (N_obs_). In comparing two competing models, Akaike proposed that if
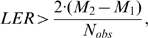
(4)model 1 should be rejected [39]. The AIC establishes a threshold for testing a hypothesis associated with model fitness. For comparing non-nested models, a Monte Carlo approach has been proposed to determine the level of significance associated with model discrimination [41].

## Supporting Information

Appendix S1 Derivation of Physiology-based Model
(0.13 MB DOC)
