# Supplementary material for: Extent of Beta Cell Destruction Is Important but Insufficient to Predict the Onset of Type 1 Diabetes Mellitus
Source: PLoS One. 2008 Jan 2;3(1):e1374. doi: 10.1371/journal.pone.0001374 (PMC2147725; doi:10.1371/journal.pone.0001374)
Supplement: Appendix S1 — Derivation of Physiology-based Model (0.13 MB DOC) [file pone.0001374.s001.doc]

Appendix S1: 
Derivation of Physiology-based Model
The rate of change in insulin in the body is a dynamic balance between a source and sinks for its removal: 
Rate of change in insulin = Source - Sinks.                                       (A-1)
This relationship can be expressed in terms of a differential equation: 
,	                                                (A-2)
where NI is the number of insulin molecules in the body, a is the maximum rate of insulin release per beta cell mass, BCMMIN(t) is the minimum required beta cell mass to maintain euglycemia, kd is the rate constant for insulin clearance from the body, CI is the plasma concentration of insulin, and V is the volume of the human body. The volume of the human body is approximated by the relation: 
,                                                                 (A-3)
where BWt(t) is the body weight and r is the average density of the human body. Under fasting conditions, the source and sinks are exactly balanced such that the rate of change of insulin is equal to zero (i.e., dNI/dt = 0). Substituting equation A-3 into equation A-2 and rearranging the terms to solve for BCMMIN(t) gives: 
,                                                   (A-4)
By defining K to be equal to  simplifies equation A-4 to: 

,                                                          (A-5)
The total beta cell mass (BCMTotal(t)) can be represented as the sum of the minimum beta cell mass and excess, or reserve capacity, beta cell mass (BCMExcess(t)): 

,                                              (A-6)
Equation A-6 can be rearranged and combined with equation A-5 to solve for the normalized excess beta cell mass (EBCM(t)): 
                                        (A-7) 
Equation A-4 can also be rearranged to solve for the concentration of insulin in the body: 

                                                          (A-8)
